# Quality of Life and Functional Outcomes in Young Women After Pelvic Fracture Fixation: A Clinical Study and Literature Review

**DOI:** 10.3390/jcm15083032

**Published:** 2026-04-16

**Authors:** Emmanuele Santolini, Amit Davidson, Kathryn Lowery, Nikolaos K. Kanakaris, Peter V. Giannoudis

**Affiliations:** 1 Orthopaedics and Trauma Unit, Emergency Department, IRCCS Ospedale Policlinico San Martino, 16132 Genova, Italy; 2 Academic Department of Trauma and Orthopaedics, School of Medicine, University of Leeds, Leeds LS1 3EX, UKkathryn.lowery1@nhs.net (K.L.); n.kanakaris@nhs.net (N.K.K.);; 3 Trauma and Orthopaedics Department, Shaare Zedek Medical Centre, Jerusalem 9103102, Israel; 4 NIHR Leeds Biomedical Research Centre, Chapel Allerton Hospital, Leeds LS7 4SA, UK

**Keywords:** pelvis, pelvic fracture, pelvic ring injury, young, female, woman, women, gender, PROMs

## Abstract

**Background**: Pelvic fractures in young patients are typically associated with high-energy trauma and long-term functional impairment. Young women may experience additional gender-specific sequelae following surgical treatment. This study aimed to evaluate functional outcomes and quality of life in young women following pelvic ring injuries. **Methods**: A retrospective cohort study was conducted including female patients of childbearing age (16–45 years) who sustained pelvic ring injuries and underwent surgical treatment at a single Level 1 trauma centre between 2009 and 2019. Validated PROMs were used to assess quality of life (EQ-5D, EQ-5D-VAS), and functional outcomes (Majeed Pelvic Score), along with a self-designed questionnaire to collect obstetric-related data. Radiographic measurements were performed to assess fracture reduction. PROM results were analysed descriptively and correlations between outcome scores were assessed using Pearson correlation. **Results**: A total of 32 patients completed all questionnaires and were included in the analysis. The mean EQ-5D index score was 0.61 (SD 0.31), the mean EQ-5D-VAS score was 68 (SD 24), and the mean Majeed Pelvic Score was 77 (SD 21). Most patients achieved good or excellent functional outcomes according to the Majeed score. Strong correlations were observed between PROM scores. Obstetric follow-up data were available for 21 patients; among these patients, 53% reported fear of pregnancy following the injury. **Conclusions**: Young women treated surgically for pelvic ring injuries demonstrated generally good pelvic-specific functional outcomes but lower quality-of-life scores compared with population norms. Obstetric concerns were commonly reported. Larger prospective studies are required to better understand long-term outcomes in this patient population.

## 1. Introduction

In recent years, there has been an increasing interest within the orthopaedic community in documenting global functional outcomes as well as quality of life scores following major trauma [1,2]. In this context, patient-reported outcome measures (PROMs) have become essential tools for guiding and targeting treatment strategies, enabling surgeons to assess treatment efficacy. Cultural and demographic factors influence PROMs, and gender-related differences may also play a role [3,4,5,6].

Pelvic fractures at a young age typically result from high-energy trauma and are associated with long-term morbidity affecting patients’ functional status and quality of life [7]. Following pelvic ring reconstruction, in addition to physical and psychological sequelae, young female patients may experience gender-specific long-term outcomes related to pregnancy, delivery method, sexual and urogenital function [8,9,10,11,12,13].

Notably, studies investigating PROMs in female patients with pelvic fractures are limited and predominantly based on non-operatively treated patient cohorts [8,12,13]. The impact of age, radiographic measurements (pelvic displacement and symmetry), hardware removal, and pre- and post-injury parity status on patients’ outcomes remains poorly explored. Operatively treated patients for pelvic ring injuries generally sustain high-energy trauma with multiple associated injuries and face unique functional and rehabilitative challenges. A better understanding of PROMs in this specific patient population would therefore enable clinicians to contextualise outcomes and compare them with reported reference values more accurately.

Although previous studies evaluating surgically treated pelvic fractures have identified factors such as fracture severity, age and associated injuries as determinants of PROMs, the impact of radiographic measurements, hardware removal, and parity status before and after injury remains underexplored [7,12]. A comprehensive evaluation on how these specific factors influence the outcomes of young women treated operatively would significantly improve the decision-making process for this population.

This study aimed to evaluate functional outcomes and quality of life in young women undergoing surgical fixation for pelvic fractures and to describe clinical factors potentially associated with patient-reported outcomes.

## 2. Materials and Methods

Following approval by the Institutional Review Board, data were collected on young women of childbearing age (16–45 years at the time of injury) who sustained severe pelvic ring injuries and were treated at a single Level 1 trauma centre, between 2009 and 2019. Although the inclusion criteria allowed patients aged 16–45 years, the actual age range of the included cohort was 18–42 years. The study included female patients who underwent operative treatment for pelvic fractures. Patients who died during the initial hospitalisation were excluded. Patients who did not complete all questionnaires or for whom complete medical or imaging records were unavailable were also excluded. Only patients with a minimum follow-up of one year were included in the analysis.

Eligible patients were identified retrospectively through hospital electronic medical records. Patient demographics, mechanism of injury, associated injuries, fixation methods, hardware removal and other pelvic reoperations details were extracted from the electronic hospital records.

Patient-reported outcome measures (PROMs): Initial contact was made via mail, offering patients the option to respond by email and complete the questionnaires online. Non-respondents to the initial post communication were subsequently contacted by telephone. A trained research assistant, not involved in patient treatment, conducted the telephone interviews, allowing patients to complete the questionnaires either by phone or online, according to their preference.

Participants were asked to complete the following questionnaires: EQ-5D-5L; EQ-5D-VAS; the Majeed Pelvic Score, and a self-designed questionnaire (Supplementary Materials) collecting data regarding obstetric-related concerns, pregnancy rates and delivery methods.

*Majeed Pelvic Score (MPS):* The MPS is a physician-rated score used to assess function after major pelvic ring injuries [14]. It comprises seven items across five subscales: pain, work, sitting, standing and sexual intercourse. The maximum score is 100 points, or 80 points for patients not working before the injury. These cut-off values were used as outcome measures in this study. To account for employment status at the time of injury, a multiple-choice question (“Work status at the time of injury: no regular work or regular work”) was included. To enable comparability between the two patient groups, MPS values were normalised to a 100-point scale by proportionally converting scores from patients with a maximum score of 80.

*EuroQuol-5D (EQ-5D):* The EQ-5D is a 5-item questionnaire that measures health-related quality of life based on five dimensions of health: mobility, self-care, daily activities, pain/discomfort and anxiety/depression [15]. The EQ-5D is widely used and is both valid and reliable across a multitude of conditions. In addition, the published EQ-5D results for average females from the same country and age range were used for comparison.

Radiographic Analysis: Using the institutional Picture Archiving and Communication System (PACS), patients’ radiographs and CT scans obtained at hospital presentation were independently reviewed by two individual orthopaedic surgeons and authors (A.D, E.S.). Pelvic fractures were classified according to the Young & Burgess and AO/OTA classifications. In cases of disagreement between the examiners, images were re-evaluated until consensus on fracture classification was achieved.

**Figure 1:**
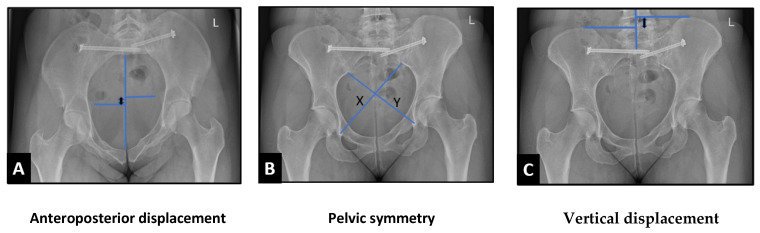
Illustration demonstrating the displacement measurements measured in different views. (**A**) Anteroposterior (AP) displacement measured on inlet radiographs as the distance between perpendicular lines drawn from the pelvic midline to each ischial spine; (**B**) Pelvic symmetry assessed on AP radiographs according to Lefaivre et al. [17], calculated as the difference between distances from the inferior aspect of the sacroiliac joint to the contralateral teardrop (X–Y). (**C**) Vertical displacement measured according to Henderson et al. [16], defined as the distance between horizontal lines drawn through the most superior points of each iliac wing.

Postoperative radiographs evaluated fixation quality. Radiographic measurements were performed on anteroposterior (AP), Inlet, and outlet radiographs obtained at a minimum follow-up of 1 year after the injury. Anteroposterior displacement was evaluated on inlet x-rays, while vertical displacement was measured according to Henderson et al. [16], by drawing perpendicular lines from the most proximal point of the ilium on each side to the pelvic midline. Pelvic symmetry was assessed on AP radiographs according to the criteria of Lefaivre et al. [17], measuring the distance from the inferior aspect of the sacroiliac joint to the contralateral teardrop. In addition, post-surgical residual fracture displacement was evaluated according to Matta’s criteria [18]. Patients were categorised into two groups based on displacement severity: those with displacement >10 mm in one or more radiographic measurements were assigned to the displacement group; those with displacement ≤10 mm were assigned to the comparison group (Figure 1).

Statistical Analysis: Data were analysed using SPSS software (version X). Continuous variables are presented as means with standard deviations and ranges, while categorical variables are presented as counts and percentages. Correlations between outcome scores (EQ-5D, EQ-5D-VAS and Majeed Pelvic Score) were assessed using Pearson’s correlation coefficient. Statistical significance was set at *p* < 0.05.

## 3. Results

Of 76 female patients who met the inclusion criteria, 32 completed all questionnaires and served as the basis for this study. Among the remaining patients, ten refused to participate, one did not speak English, and the remaining patients were uncontactable despite multiple attempts, including personal telephone calls, mailed letters to recorded addresses, and contact with their general practitioners.

Table 1 summarises the demographics, follow-up time, associated injuries, mechanism of injury, pelvic fracture classification, fixation modality, and postoperative radiographic displacement measurements of the 32 patients included in the study. Of these, seven patients had a displacement greater than 10 mm in at least one of the radiographic measurements of interest.

**Figure 2:**
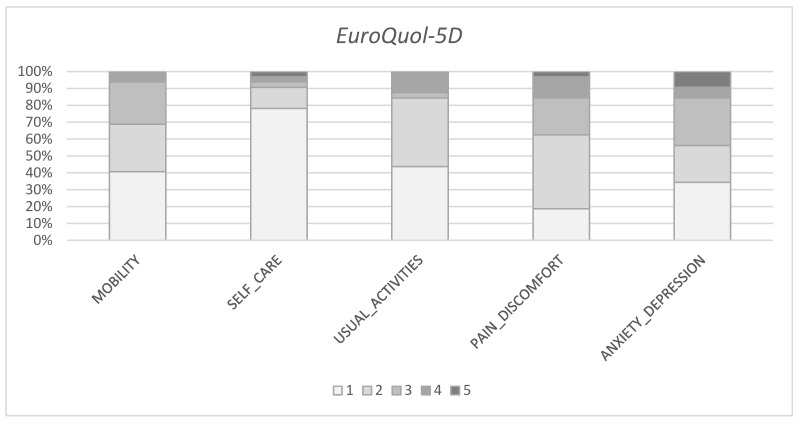
Distribution of responses across the five EQ-5D domains. The proportion of participants is shown for each domain and represented by different colours according to the EQ-5D response levels, ranging from 1 (no problems) to 5 (extreme problems).

With regard to PROMs score, the mean EQ-5D index score was 0.61 (SD 0.31) and the mean EQ-5D-VAS score was 68 (SD 24). The mean Majeed pelvic score was 77 (SD 21). Domain-specific EQ-5D results and MPS clinical grade distribution are presented in Figure 2 and Table 2.

Complete obstetric follow-up data were available for 21 patients. Among these patients, a total of 20 deliveries occurred after the injury, including 11 vaginal deliveries and 9 caesarean sections. Overall, eight patients (25% of the study cohort) had children after the pelvic injury. The most commonly reported reason for caesarean section was a recommendation by the obstetrician (four patients), whereas three patients reported personal preference. In two cases, other obstetric indications unrelated to the pelvic injury were cited as the reason for caesarean delivery. In the available medical records, no caesarean section was explicitly documented as being performed due to concerns related to the previous pelvic fracture. Additionally, 53% of patients who completed the questionnaire reported being afraid of becoming pregnant following their injury (Table 1).

Correlation analysis of the EQ-5D, EQ-5D-VAS, MPS, and MPS cut-off values revealed strong correlation among all PROMs (Table 3).

## 4. Discussion

In this study, PROM results of young females who sustained operative treatment for pelvic fracture are presented. PROMs chosen were EQ-5D, EQ-5D-VAS and MPS. The EQ-5D values were below population norms, and both EQ-5D and MPS scores were at the lower end of previously reported PROMs for patients investigated after pelvic fractures.

The mean EQ-5D (0.61) and EQ-5D-VAS (67.9) scores observed in our study were lower than the recently reported national population norms for comparable age groups, which were 0.934–0.905 and 86.5, respectively [19]. Previous reports on EQ-5D values after pelvic fractures have ranged from 0.63 to 0.8 [7]. Our results fall within the lower range of previously reported PROMs values. This may be explained by the fact that most previous studies reporting on PROMs of patients with pelvic trauma included individuals across all age groups and patients treated non-operatively. Moreover, gender could have influenced the results, with a negative impact on PROMs scores in women suffering from multiple trauma, as reported by Holbrook et al. [6] and more recently by Banierink et al. [20]. Interestingly, although most patients achieved good or excellent functional outcomes according to the Majeed Pelvic Score, the EQ-5D values remained lower than population norms. This suggests that pelvic-specific functional recovery may be satisfactory while broader aspects of health-related quality of life may remain affected following high-energy pelvic trauma.

The results of previous studies which investigated outcomes in young women following pelvic trauma [8,12,13], are summarised in Table 4. Similarly to our findings, all of these studies reported lower PROMs values when compared to population norms, despite the inclusion of non-operatively treated patients. A direct comparison with our results is not feasible, as different outcome measures were used and none of them employed EQ-5D or the MPS.

Majeed’s pelvic score is the most commonly used injury-specific questionnaire for patients with pelvic fractures [7,21]. In the present study, it was used in addition to the EQ-5D to combine generic and disease-specific instruments allowing the assessment of condition-specific outcomes, without missing important determinants of an individual’s health state. In our study, almost 80% of patients showed excellent or good clinical outcomes (Table 2) with a mean Majeed Pelvic Score of 76 (SD 14) which lies at the lower end of previously reported values (70–95) [7]. The same assumptions made for the EQ-5D results may also explain why our study population showed lower MPS scores. Although very commonly used, concerns regarding the accuracy and consistency of reports employing the MPS have been raised [22]. Indeed, direct comparison of our questionnaire scores to previously reported data is not straightforward. The main source of inconsistency is that most previous reports did not distinguish between working from and non-working patients at the time of injury which affect the scores. To enable comparison with earlier reports, we extrapolated scores for non-working patients as explained in the materials and methods section, as previous authors provided a single mean score for both groups [22,23].

Given the limited sample size, multivariable regression analysis was not performed and the study should be interpreted as descriptive.

The relatively high Injury Severity Score of the cohort (mean ISS 15) indicates that many patients sustained multisystem trauma. Therefore, PROM outcomes likely reflect the combined effect of pelvic injuries and associated trauma rather than the pelvic fracture alone.

The relationship between radiographic reduction quality and patient-reported outcomes remains controversial. In the present study, seven patients demonstrated displacement greater than 10 mm in at least one radiographic plane; however, no clear association with PROMs was observed. These findings should be interpreted cautiously given the limited number of patients with significant displacement. Accordingly, the relationship between functional outcome and reduction quality remains debatable, as also reported by Therrien et al. [24] who investigated the same correlation in a cohort of 130 patients with pelvic ring injuries at 1-year follow-up and found no or only weak correlation between functional status and pelvic symmetry.

Hardware removal was performed in only three patients in our cohort; therefore, no meaningful analysis regarding its influence on PROMs could be performed. These observations should therefore be interpreted as descriptive findings rather than analytical results.

Obstetric findings in this study should be interpreted as descriptive observations due to the limited number of patients with available pregnancy data. Nevertheless, an important finding was that 53% of participants reported fear of pregnancy after pelvic injury. This observation highlights a potentially relevant psychosocial concern that may influence reproductive decisions in young women following pelvic trauma.

We observed a strong correlation among the PROMs scores that formed the basis of this study (Table 3). Other authors have reported a correlation between PROMs and functional status in patients suffering from pelvic fractures [25,26] or with the physical domain of the generic SF-36 questionnaire only [27]. To our knowledge, this is among the first studies to explore the relationship between MPS and EQ-5D in this population. Our findings indicate that meaningful comparisons can be made between studies reporting these widely used PROMs for patients with pelvic injuries.

The main limitation of this study is the relatively low number of participants, which was mitigated to some extent by the administration of multiple PROMs to each included participant. Furthermore, although many patients were eligible, many were either unavailable or declined to participate in the study. However, the vast majority of these patients were people who had relocated to different regions, which may also have introduced selection bias in our study. Therefore, due to the limited cohort size and response rate, the present study should primarily be interpreted as a descriptive cohort study rather than a predictor-analysis study.

Young women treated surgically for pelvic ring injuries demonstrated generally good pelvic-specific functional outcomes but lower quality-of-life scores compared with population norms. These findings should be interpreted in the context of associated injuries, as many patients sustained multisystem trauma. Obstetric concerns, particularly fear of pregnancy, were commonly reported. Larger multicentre prospective studies are required to better understand long-term outcomes and reproductive implications in this patient population.

## Figures and Tables

**Table 1:** Demographic characteristics, associated injuries, mechanism of injury, fracture classification, fixation modality, postoperative radiographic displacement measurements, and obstetric-related data of the study cohort.

Number of patients	32
Age [years] (mean, range)	25 (18–42)
Follow-up [years] (mean, range)	7.3 (3–14) (SD 4.2)
*Fracture classification*	
LC 1	19
LC 2	2
LC 3	5
APC 2	1
APC 3	3
VS	2
*Mechanism of injury*	
Motor vehicle collision	17
Pedestrian versus car	3
Fall from Height	8
Other	4
*Associated injures*	
ISS score, mean (range, SD)	15 (34–9, 7.2)
*Associated injuries, No. patients*	
Head injury	9
lower extremity	10
upper extremity	7
Chest	4
Abdominal	8
Bladder	1
Spine	10
*Fracture fixation modality*	
Posterior fixation	
Sacroiliac screw	31
Anterior fixation	
External fixation	17
Retropubic screw	4
Plate	4
Hardware removal	3
Time from initial operation to hardware removal [months] (mean, range)	20 (11–41)
*Obstetrics*	
Patients with Children before injury	8 (25%)
Patients who gave birth after the injury	8 (25%)
Hardware removed in patient who had children	1
*Radiographic measurements, mm, mean (range, SD)*	
Pelvic asymmetry	6.9 (20–0,6)
Vertical displacement	3 (11–0, 2.8)
Anteroposterior displacement	4.2 (14–0, 3.35)

**Table 2:** Results of the EQ-5D and Majeed Pelvic Score (MPS) questionnaires. EQ-5D results are presented across the five domains and categorised into three levels based on patient responses: no problems (responses 1–2), moderate problems (response 3), and severe problems (responses 4–5). The MPS clinical grade, as defined by Majeed, and the mean score are also reported.

EQ-5D-Questionnaire	
Mobility	
No problems (%)	22 (69%)
Moderate problems (%)	8 (25%)
Severe problems, (%)	2 (6.3%)
Self-care	
No problems (%)	29 (91%)
Moderate problems (%)	1 (3%)
Severe problems (%)	4 (6%)
Usual activities	
No problems (%)	27 (84%)
Moderate problems (%)	1 (3%)
Severe problems, (%)	4 (13%)
Pain/Discomfort	
No problems (%)	20 (63%)
Moderate problems (%)	7 (22%)
Severe problems (%)	5 (16%)
Anxiety/Depression	
No problems (%)	18 (56%)
Moderate problems (%)	9 (28(%)
Severe problems (%)	5 (16%)
EQ-5D Index, mean (SD)	0.61 (0.31)
EQ-VAS, mean (SD)	68 (24)
**Majeed Questionnaire**	
Average score (SD)	77 (21)
Clinical grade, No. of patients	
Excellent	12 (38%)
Good	12 (38%)
Fair	2 (6%)
Poor	6 (18%)

**Table 3:** Correlation between EQ-5D outcomes and the Majeed Pelvic Score.

	Pearson Test	Sig. (2Tailed)	95% Confidence Intervals (2-Tailed) ^a^	
			Lower	Upper
EQ-5D Index—Majeed pelvic score	0.790	**<0.001**	0.609	0.893
EQ-5D Index—Majeed score Cat	0.739	**<0.001**	0.525	0.865
VAS EQ-5D—Majeed pelvic score	0.683	**<0.001**	0.438	0.833
VAS EQ-5D—Majeed score Cat	0.586	**<0.001**	0.299	0.776
EQ-5D Index—VAS EQ-5D	0.769	**<0.001**	0.575	0.881

^a^ Estimation is based on Fisher’s r-to-z transformation.

**Table 4:** Summary of studies reporting patient-reported outcome measures (PROMs) in female patients with pelvic fractures. The number of female patients, age, surgical treatment, PROMs used, and main conclusions are presented.

Article	Number of Patients	Age at Injury-Mean (Range)	Age at PROMS-Mean (Range)	Number of Surgically Treated Patients	PROM Used	Conclusions
Vallier et al. (2012) [12]	87	33.5 (16–63)	Not specified	49 out of 87 women surgically treated	Musculoskeletal Functional Assessment (MFA)	Wide variability in functional outcomes with substantial residual dysfunction; improved outcomes in isolated fractures without lower extremity injuries; poorer MFA scores associated with bladder rupture and anteroposterior compression injuries.
Cannada et al. (2010) [8]	71	27.8 (17–44)	33.8 (19–55)	40 out of 66 women surgically treated	SF-12	Functional outcomes were associated with fracture pattern and surgical treatment; women who had children post-injury demonstrated higher overall SF-12 scores.
McCarthy et al. (1995) [13]	233 (123 pelvic fracture; 110 lower extremity fracture)	27.2	31.5	Not specified	SF-36	Women scored lower than population norms across all SF-36 domains except mental health; high rates of reduced self-perceived attractiveness and decreased sexual pleasure were reported post-injury; comorbid chronic conditions were significant predictors of poorer health outcomes.
Current study	32	25 (18–42)	32 (19–53)	32	EQ-5D-5L; EQ-5D-VAS; Majeed pelvic questionnaire and a self-designed questionnaire	

## Data Availability

The raw data were generated from electronic medical records of the participating medical centre and from questionnaires completed by the patients. The derived data supporting the findings of this study are available from the corresponding author (E.S.) upon request.

## Supplementary Materials

The following supporting information can be downloaded at: https://www.mdpi.com/article/10.3390/jcm15083032/s1, File S1: Self-Designed Questionnaire on Obstetric-Related Concerns, Pregnancy Rates, and Delivery Methods.
