# Supplementary material for: Quality of Life and Functional Outcomes in Young Women After Pelvic Fracture Fixation: A Clinical Study and Literature Review
Source: J Clin Med. 2026 Apr 16;15(8):3032. doi: 10.3390/jcm15083032 (PMC13116550; doi:10.3390/jcm15083032)
Supplement: Supplementary file 1 [file jcm-15-03032-s001.zip › jcm-4192917-supplementary.pdf]

Name____________ **NHS number_____________** **Date of birth _______**

Date of completion of the questionnaire _________

Questionnaire - pregnancy and delivery

1. Were you ever pregnant *before* your pelvic injury? **YES** **NO**

If yes,

Number of pregnancies _____

what have been the outcomes of your pregnancies:

Vaginal delivery _______

Forceps/vacuum delivery _______

C-section _______

Miscarriage _______

Ectopic/Termination _______

Please provide detailed information:

2. Have you been pregnant *after* your pelvic injury? **YES** **NO**

If yes,

Number of pregnancies _____

what have been the outcomes of your pregnancies:

Vaginal delivery _______

Forceps/vacuum delivery _______

C-section _______

Miscarriage _______

Ectopic/Termination _______

Please provide detailed information:

Have you had an attempt of vaginal delivery?

what have been the outcomes of this attempt (i.e. Cesarean Section, Normal Vaginal Delivery, Termination and or Miscarriage)?

Have you been advised to have a C section since your pelvic injury? YES NO

If Yes By whom you were recommended to do the C section and why?

3. Have you had the metal work inserted in your pelvis removed after the injury: **YES** **No**

If yes how long after the injury was removed (years/months):

4. Have you desired to be pregnant *since your pelvic injury*? **YES** **NO**

5. Are you afraid to become pregnant *since your pelvic injury*? **YES** **NO**

6. Have you been advised not to become pregnant *since your pelvic injury*? **YES** **NO**

If yes, By whom you were advised not to become pregnant and why?

Additional information:
